# An Efficient Electronic Cash System Based on Certificateless Group Signcryption Scheme Using Conformable Chaotic Maps

**DOI:** 10.3390/s21217039

**Published:** 2021-10-23

**Authors:** Chandrashekhar Meshram, Agbotiname Lucky Imoize, Amer Aljaedi, Adel R. Alharbi, Sajjad Shaukat Jamal, Sharad Kumar Barve

**Affiliations:** 1 Department of Post Graduate Studies and Research in Mathematics, Jaywanti Haksar Govt. Post-Graduation College, College of Chhindwara University, Betul 460001, India; cs_meshram@rediffmail.com; 2 Department of Electrical and Electronics Engineering, Faculty of Engineering, University of Lagos, Akoka, Lagos 100213, Nigeria; 3 Department of Electrical Engineering and Information Technology, Institute of Digital Communication, Ruhr University, 44801 Bochum, Germany; 4 College of Computing and Information Technology, University of Tabuk, Tabuk 71491, Saudi Arabia; aaljaedi@ut.edu.sa (A.A.); aalharbi@ut.edu.sa (A.R.A.); 5 Department of Mathematics, College of Science, King Khalid University, Abha 61413, Saudi Arabia; shussain@kku.edu.sa; 6 Water Resources and Applied Mathematics Research Lab, Nagpur 440027, India; drshardbarve@rediffmail.com

**Keywords:** certificateless group signcryption scheme (CGSS), conformable chaotic maps (CCM), electronic cash system (ECS), signcrypter, provably secure schemes, authentication, E-commerce channels

## Abstract

Signcryption schemes leveraging chaotic constructions have garnered significant research interest in recent years. These schemes have proffered practical solutions towards addressing the vast security vulnerabilities in Electronic Cash Systems (ECS). The schemes can seamlessly perform message confidentiality and authentication simultaneously. Still, their applications in emerging electronic cash platforms require a higher degree of complexity in design and robustness, especially as billions of online transactions are conducted globally. Consequently, several security issues arise from using open wireless channels for online business transactions. In order to guarantee the security of user information over these safety-limited channels, sophisticated security schemes are solely desired. However, the existing signcryption schemes cannot provide the required confidentiality and authentication for user information on these online platforms. Therefore, the need for certificateless group signcryption schemes (CGSS) becomes imperative. This paper presents an efficient electronic cash system based on CGSS using conformable chaotic maps (CCM). In our design, any group signcrypter would encrypt information/data with the group manager (GM) and send it to the verifier, who confirms the authenticity of the signcrypted information/data using the public criteria of the group. Additionally, the traceability, unforgeability, unlinkability, and robust security of the proposed CGSS-CCM ECS scheme have been built leveraging computationally difficult problems. Performance evaluation of the proposed CGSS-CCM ECS scheme shows that it is secure from the Indistinguishably Chosen Ciphertext Attack. Finally, the security analysis of the proposed technique shows high efficiency in security-vulnerable applications. Overall, the scheme gave superior security features compared to the existing methods in the preliminaries.

## 1. Introduction

In modern electronic commerce, digital signatures play a significant role due to integrity and authentication requirements. Integrity is a vital property that helps to monitor the received messages from being modified by an adversary, while the authentication property helps protect the sender from impersonation [1]. Currently, group signcryption schemes are gaining entrance into the e-commerce space. For example, Chaum and van Heyst [2] introduced a group signature scheme that allows a signature from any group member to represent the group. However, several limitations of group signature schemes have been identified [3,4,5]. Only group members are eligible to sign, and the message receiver cannot know the signer, among others. In practice, a signcryption scheme should be designed to meet specific security attributes such as public verifiability, ciphertext authentication, public ciphertext authentication, and ciphertext anonymity [3,4,5,6,7]. Under favorable conditions, these should be designed with extremely hard assumptions. However, if an adversary can solve the hardness assumption of a given signcryption scheme, they can conveniently process the private keys of each user in the system [8]. The ability of the foe to solve the hardness assumption poses a severe security threat in electronic commerce channels, and the need to address such security vulnerabilities is not negotiable. In order to address this problem, this paper presents an efficient electronic commerce system based on a certificateless group signcryption scheme (CGSS) using conformable chaotic maps (CCM).

### 1.1. Contributions

The contributions of the paper are outlined as follows. First, we give comprehensive literature on the electronic commerce system based on certificateless group signcryption schemes. To ensure consumers’ anonymity in e-commerce platforms, we merged the valuable features of a certificateless signature scheme (CSS) with a group signcryption scheme (GSS) in the projected CGSS-CCM scheme for the electronic cash system (ECS). This study proposed a new efficient certificateless group signcryption scheme and electronic cash system (ECS). For the electronic cash system development, we used certificateless group signcryption schemes, and for the development of certificateless group signcryption schemes, we used conformable chaotic maps. A group signcrypter, with the help of the group manager (GM), encrypts a communication on behalf of the group in our design. In this scenario, any group signcrypter would encrypt information/data with the GM and have it sent to the verifier, who then approves the authenticity of the signcrypted information/data using the public criteria of the group. We further examined the proposed scheme’s security to confirm that neither the GM nor any other group member can yield a legal signcrypted text. Additionally, we carried out a performance analysis of the proposed CGSS-CCM scheme and demonstrated its indistinguishably under the chosen ciphertext attack. The traceability, unforgeability, unlinkability, and robust security of the proposed CGSS-CCM ECS scheme were verified using computationally difficult problems. Finally, we compared the security features of the proposed CGSS-CCM ECS scheme with the existing techniques using several standard metrics.

### 1.2. Paper Organization

The organization of the paper is as follows. Section 2 covers the related works. Section 3 presents the background and material. Section 4 covers the proposed certificateless group signcryption scheme using conformable chaotic maps; this section also captures the setup, partial private key generation, private key generation. user key generation, signcryption, verification, and opening. Section 5 gives a detailed security investigation of the proposed CGSS-CCM ECS scheme. In Section 6, the proposed electronic cash system based on CGSS using conformable chaotic maps is detailed. The scheme comprises the initialization, joining, withdrawal, payment, deposit, and identity revocation phases. In Section 7, the security analysis comprising unforgeability and anonymity of the proposed ECS scheme is highlighted, and the efficiency of the scheme is demonstrated. Section 8 focuses on the performance comparison of the proposed CGSS-CCM with other related schemes. Finally, Section 9 provides a concise conclusion to the paper.

## 2. Related Works

Conformable chaotic maps (CCM) are used to generate public and secret parameters of the proposed CGSS. CCM and pairing are different; the development of CCM depends on the Chebyshev polynomial, but pairing depends on the bilinear pairing operation of the function. Pairing operations cover more computation costs than chaotic maps or CCM. Therefore, CCM or chaotic maps play a significant role in developing the lightweight cryptographic scheme compared to pairing operation.

In the existing literature, Shamir [3] reported an identity-based cryptographic scheme, whose idea motivated an identity-based multi-signcryption scheme [4], and a certificateless signature without pairing [5]. Similarly, Park et al. [6] reported an identity-based group signature, which allows verification of the group signature by examining the identities of the group members. However, if a change in the group structure occurs, previous group signatures provided by other group members become invalid. But this limitation is undesirable in practical e-commerce systems. Tseng and Jan [7] presented a related ID-based scheme that addresses most of the flaws identified in Park et al. [6]. In several works of literature, the key escrow problem has been named one of the main flaws of ID-based cryptosystems. Al-Riyami and Paterson [8] reported an encryption scheme that does not need a public key to address this issue. Similarly, Ma, Ao, and He [9] proposed a certificateless group signature to address the key escrow problem in ID-based group signature schemes.

In recent years, public-key cryptosystems are fast gaining widespread popularity in guaranteeing message confidentiality, non-repudiation, and more. Firstly, the message that has the private key of the sender is signed, and the message signature pair is encrypted using a temporal session key [10,11]. Consequently, the receiver’s public key can be used to encrypt the session key before transmission, and the session key retrieved by the receiver recovers sent messages using his private key. This procedure is carried out after both the random session key and the receiver get the encrypted message-signature pair [12]. Afterwards, the receiver decrypts the encrypted message-signature pair using the session key. In this case, the authenticity and integrity of the message are confirmed by the receiver by verifying the signature using the sender’s public key. However, the traditional signature and then encryption technique is cost-prohibitive and computationally intensive. In order to decrease the cost and processing time of this scheme, the idea of signcryption that combines the features of digital signature and encryption is presented by Zheng [13].

The signcryption scheme reported by Zheng uses the discrete logarithm (DL) problem over a finite field. Interestingly, an enhanced form of Zheng’s scheme had been reported by Zheng and Imai [14] to tackle the inherent public verifiability issues discovered in the scheme reported by Zheng [13]. In the same vein, Bao and Deng’s [15] modification to Zheng’s scheme allows public verifiability. However, public verifiability is undesirable in practical applications requiring firewall filtering [16].

Gamage et al. [17] reported a robust signcryption scheme that maintains the public ciphertext authentication property. The scheme allows a seamless signature verification without an external entity based on the computationally Diffie–Hellman (CDH) protocol [18]. However, the CDH-based protocol cannot perform ciphertext anonymity. Consequently, a foe can conduct random checks to decipher the message’s originality [19]. In practice, this is not desirable in e-commerce, where there is a need to adequately preserve the sender’s information from any adversary. However, the schemes mentioned above did not address the forward secrecy property, which is crucial in e-commerce. Motivated by this gap in the literature, Chow et al. [19] offered a forward secure signcryption scheme that allows public ciphertext authentication. However, the scheme uses bilinear pairing, which increases the computational complexity [20].

Han et al. [21] have provided a forward secrecy scheme that does not use bilinear pairing. The scheme shows better efficiency than Chow et al. [19]. A forward secure proxy signcryption scheme with public verifiability was presented by Elkamchouchi, Nasr, and Ismail [22]. Though this scheme aggregates hard problems, it showed limited efficiency, perhaps due to composite modulus design, and cannot perform ciphertext authentication. Additionally, Iqbal and Afzal [23] have reported a related construction with forward secrecy and public ciphertext authentication for several applications. In a related study, Chaudhry et al. [24] offered a signcryption scheme tailored for an e-commerce system. Still, the protocol cannot support forward secrecy and public verifiability, which are candidate requirements in e-cash systems [25,26].

The security of electronic cash systems is a significant issue contending the rapid development of e-commerce. Several security schemes have been presented to tackle this issue [27,28]. Specifically, Wang, Cao and Zhang [27] offer a novel scheme for untraceable electronic cash transactions based on discrete logarithm assumption and the cut-and-choose approach. Here, the bank is not involved in any payment between a user and a receiver. 

In [28], the authors utilized the concept of a group signature scheme to design a robust ECS. However, the security issues threatening e-commerce channels remain, especially as the business community is growing exponentially. Thus, the security of e-commerce platforms is ripe for comprehensive research exploitation.

Following the preceding security schemes deployed in electronic cash systems, several electronic cash protocols leveraging cryptographic constructions have been reported [29,30,31,32,33,34]. In particular, Lee, Choi, and Rhee [29] proposed a robust security scheme to address the problem of double-spending in secure electronic cash systems. In work, due to Nishide and Sakurai [30], a security scheme has been offered to secure offline anonymous electronic cash systems. The goal is to preserve sensitive user information from being compromised by insiders. Kutubi, Alam, and Morimoto [31] proposed an offline electronic payment scheme that satisfies essential security requirements of e-payment platforms was proposed. The scheme offers simple computations, and the merchant can verify the spent e-coin leveraging Schnorr’s blind signature. Additionally, the scheme enables trusted authorities to identify the dishonest spender if multiple spending occurs with ease.

Additionally, Islam [32] reported a provably secure pairingless identity-based signature scheme for use in an e-cash system. Recently, an exchange centre-based digital cash payment solution was reported by Xu and Li [33] to address several security issues proliferating the e-commerce domain. Lastly, Alidadi et al. [34] offered an identity-based signature with key revocation functions for a cloud-enabled mobile payment system.

It is evident, based on the previous research, that no work has implemented the certificateless group signcryption scheme based on conformable chaotic maps in an electronic cash system as in our proposed work.

## 3. Background and Materials

This segment reviews the various underlying concepts relating to the work before delving into the current investigation on certificateless group signcryption schemes using conformable chaotic maps (CGSS-CCM). First, a short-lived Chebyshev chaotic map implementation is presented. This is followed by a Chebyshev polynomial, conformable chaotic maps using the minimal method, and delineated a list of other techniques used in this development. A list of symbols used in the paper is provided in Table 1.

### 3.1. Chebyshev Chaotic Polynomials

The operatory of Chebyshev sequential polynomials (CSP) is investigated (see [35]). In the ʓ variation, CSP $T_{ᶇ}\left(ʓ\right)$ is a ᶇ-degree polynomial. Let the arrangement be ʓ∈[−1, 1], and ᶇ be an integer. In general, CSP reported the following:

$$T_{ᶇ}\left(ʓ\right)=\cos\left(ᶇ\times arc\;\cos\left(ʓ\right)\right),\;T_{0}\left(ʓ\right)=1,\;T_{1}\left(ʓ\right)=ʓ,\;T_{ᶇ}\left(ʓ\right)=2ʓT_{ᶇ-1}\left(ʓ\right)-T_{ᶇ-2}\left(ʓ\right);\;ᶇ\geq 2$$

Under these conditions, the functional arc cos(ᶇ) and cos(ᶇ) denoted as arc cos: [−1, 1]→[0, π] and cos: Ɍ→[−1, 1].

CSP has two fundamental properties: chaotic and semi-group properties [36,37,38,39,40].

(1) The chaotic property: The CSP map is defined as $T_{ᶇ}:\;\left[-1,\;1\right]\to \left[-1,\;1\right]\;$ with degree ᶇ >1, is a chaotic map accompanying with the (invariant density) functional $\;f^{*}\left(ʓ\right)=\frac{1}{\left(\pi \sqrt{1-{ʓ}^{2}}\right)}$ for the positive Lyapunov exponent λ=lnᶇ>0.
(2) Semi-group property: The possessions of a semi-group meet the following criteria:
  $$\begin{array}{ll} T_{\ell }\left(T_{w}\left(ʓ\right)\right)=\cos & \left(\ell arc\cos\left(\left(\cos\left(warc\cos\left(ʓ\right)\right)\right)\right)\right)\\ & =\cos\left(\ell arc\cos\left(ʓ\right)\right)\\ & =T_{w\ell }\left(ʓ\right)\\ & =T_{w}\left(T_{\ell }\left(ʓ\right)\right), \end{array}$$
  where ʓ∈[−1, 1] and ℓ and w are positive integers.

Zhang [40] showed that the semi-group property preserves the interval (−∞,+∞), which may be utilized to improve the property as tracks:

$$T_{ᶇ}\left(ʓ\right)=2ʓT_{ᶇ-1}\left(ʓ\right)-T_{ᶇ-2}\left(ʓ\right);\;n\geq 2$$

where ʓ∈(−∞,+∞) and $q_{1}$ is a large and safe prime. As a result, the property is:

$$T_{\ell }\left(T_{w}\left(ʓ\right)\right)\left(modq_{1}\right)=T_{w\ell }\left(ʓ\right)\left(modq_{1}\right)=T_{w}\left(T_{\ell }\left(ʓ\right)\right)\left(modq_{1}\right)$$

In addition, the semi-group property is retained. It is worth noting that extended Chebyshev polynomials commute under confirmation as well.

There are two assessments for Chebyshev polynomials (CP) that consider handling in polynomial time:

(1) The discrete log’s (DL) task is to invent an integer ℓ with the end goal $T_{\ell }\left(ʓ\right)=v$ given two items ʓ and v.
(2) The Diffie–Hellman problem (DHP) task is to measure the $T_{\ell w}\left(ʓ\right)$ element due to three elements ʓ, $T_{\ell }\left(ʓ\right)$, and $T_{w}\left(ʓ\right)$.

### 3.2. Conformable Chebyshev Chaotic Maps (CCCM)

Previously, the conformable calculus (CC) was known as the conformable fractional calculus (CFC) [41]. However, it puts a burden on the known properties of fractional calculus (derivatives of non-integer power). CC, in essence, is responsible for future preparation.

Assume that u is a fractional (arbitrary) number between 0 and 1. An operator u is conformable differential if and only if $\alpha^{0}$ is the self-operator and $\alpha^{1}$ is the usual difference operational. For differentiable utility, $\alpha^{u}$ is clearly conformable if and only if β = β(y).

$$\alpha^{0}\beta \left(y\right)=\beta \left(y\right),\alpha^{1}\beta \left(y\right)=\beta \prime \left(y\right).$$

Anderson et al. [41] have proposed a new formulation of CC derived from control theory to describe the performance of a proportional-differentiation controller that conforms to the error function. The following is the structure of the instruction.

**Definition** **1.** If u ϵ [0, 1] is true, then CC has in the following documentation.

$$\alpha^{u}\beta \left(y\right)=\eta_{1}\left(u,y\right)\beta \left(y\right)+\eta_{0}\left(u,y\right)\beta \prime \left(y\right),$$

where the $\eta_{1}$ and $\eta_{0}$ functions reach the limits

$$\underset{u\;\to 0}{\lim}\eta_{1}\left(u,y\right)=1,\underset{u\;\to 1}{\;\lim}\eta_{1}\left(u,y\right)=0,\;\underset{\;u\to 0}{\lim}\eta_{0}\left(u,y\right)=0,\underset{u\;\to 1}{\lim}\eta_{0}\left(u,y\right)=1.$$

In order to get the overhead description, we shall deliberate $\eta_{1}\left(u,y\right)=\left(1-u\right)y^{u}$ and $\eta_{0}\left(u,y\right)=uy^{1-u}$, or $\eta_{1}\left(u,y\right)=\frac{\left(1-u\right)}{\Gamma \left(1+u\right)}\;\mathrm{and}\;\eta_{0}\left(u,y\right)=\frac{u}{\Gamma \left(1+u\right)}$ where $\alpha^{u}\beta \left(y\right)$ is the name of the β(y) function’s conformable differential operator. As a result, the fractional tuning connections of the function and its derivative, $\eta_{1},\eta_{0}$ are always dependably.

We obtain the resulting structure by applying the notion of CC to express the polynomial $T_{ᶇ}\left(y\right)$:

Since $T_{ᶇ}^{\prime }\left(y\right)=2ᶇT_{ᶇ-1}\left(y\right)$, then $\alpha^{u}T_{ᶇ}\left(y\right)$ has the subsequent formal relationship (1)

(1) $$T_{ᶇ}^{u}\left(y\right)=\alpha^{u}T_{ᶇ}\left(y\right)=\eta_{1}\left(u,y\right)T_{ᶇ}\left(y\right)+\eta_{0}\left(u,y\right)T_{ᶇ}^{\prime }\left(y\right)$$

**Figure 1:**
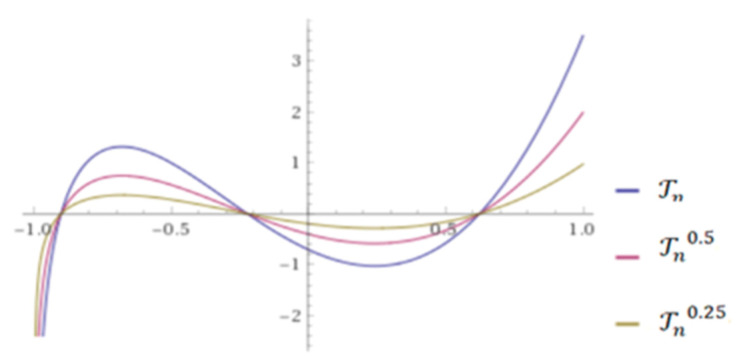
CCP for different values of u=0.25, 0.5, 1 with $\eta_{1}\left(u,y\right)=\frac{\left(1-u\right)}{\Gamma \left(1+u\right)}\;\mathrm{and}\;\eta_{0}\left(u,y\right)=\frac{u}{\Gamma \left(1+u\right)}$.

The Formula (1) can be replaced by (2)

(2) $$T_{ᶇ}^{u}\left(y\right)=\eta_{1}\left(u,y\right)T_{ᶇ}\left(y\right)+2ᶇ\eta_{0}\left(u,y\right)\times \omega \left(y\right)T_{ᶇ-1}\left(y\right),$$

where $\omega \left(y\right)=1+2y+\left(4y^{2}-1\right)+\ldots +\left(ᶇ-1\right)$-times. The conformable Chebyshev polynomials (CCP) are defined by Equation (2) (See Figure 1 [42]).

**Properties of CCCM:** The CCCM possesses the following two exciting features:

**Definition** **2.** *(Chaotic properties of CCCM)*. The CCCM satisfies recurrent relations under the chaotic property [42] i.e.,

$$T_{ᶇ}^{u}\left(y\right)=[2y\;\eta_{1}\left(\alpha ,y\right)+2ᶇ\;\eta_{0}\;\left(u,y\right)\times \omega \left(y\right)]T_{ᶇ-1}\left(y\right)-\eta_{1}\left(u,y\right)T_{ᶇ-2}\left(y\right)$$

**Definition** **3.** (Semi-group properties of CCCM). The semi-group properties look for CCCMs located on the interval (−∞, ∞) [42], i.e., $T_{k}^{u}\left(T_{ᶇ}^{u}\left(y\right)\right)=T_{ᶇ}^{u}\left(T_{k}^{u}\left(y\right)\right)=T_{kᶇ}^{u}\left(y\right)$

It is worth noting that when we use u→0, we get the original instance from [40].

At this point, we note that the DL and assignments for the CCP are approximately DHP occur.

## 4. The Proposed Certificateless Group Signcryption Scheme Based on Conformable Chaotic Maps

In this section, we introduced an efficient CGSS using conformable chaotic maps. A group of signcrypters $\left(SG:\;C_{1},C_{2},\ldots ,C_{n}\right)$ is included in the proposed CGSS-CCM, and anyone can signcrypt a message using the GM on behalf of a KGC and the group. The proposed CGSS-CCM is divided into six phases, as follows:

### 4.1. Setup

Using the safe prime techniques [43,44], the KCG chooses an integer $n=p_{1}\times p_{2}$ where $p_{1},p_{2}$ are enormous primes. After that, they choose g as a GF $\left(p_{1}\right)$ generator and pick the a∈[0,1] rational number. Then they give n and g to the GM.

### 4.2. Partial Private Key Generation (PPKG)

The KGC is in charge of this operation. As it secretes factor and their identification $TD_{\mathrm{KGC}}$, the KGC selects a master secret key $m_{sk}$ at this point. Then they assess $m_{pk}$, a public constraint whose security is guaranteed by solving conformable chaotic maps.

$$m_{pk}=T_{m_{sk}}^{a}\left(g\right)\left(mod\;n\right)$$

Then they hand over $\left(m_{pk},\;TD_{\mathrm{KGC}}\right)$ to the GM.

### 4.3. Private Key Generation (PKG)

The PKG measurements are as follows: the GM selects three private variables λ, ⅾ and $\;TD_{\mathrm{GM}}$, and then calculates the group’s public and private keys as follows.

$$G_{prk}=\lambda \times m_{pk}+TD_{\mathrm{KGC}}\times TD_{\mathrm{GM}}\left(mod\;n\right)\;G_{pbk}=T_{G_{prk}}^{a}\left(g\right)\left(mod\;n\right)\;ed\equiv 1\;mod\;\phi \left(n\right).$$

The GM then makes $\left(n,g,m_{pk},TD_{\mathrm{GM}},e,G_{prk}\right)$ variables public while keeping them $\left(\lambda ,\;e,\;G_{prk}\right)$ secret as their private key.

### 4.4. User Key Generation (UKG)

The signcrypter and the GM are in this phase. This level’s steps are listed below.

Step 1. After determining the public factor, any signcrypter picks a secret parameter $W\in {𝕫}_{n}^{*}$ on behalf of the party and calculates $\;TD_{C}$ as follows:

$$TD_{C}=T_{W}^{a}\left(TD_{\mathrm{GM}}\right)\left(mod\;n\right)$$

The $\;TD_{C}$ is then sent to the GM through a private channel.

Step 2. Following the estimation of $TD_{C}$, the GM selects a secrete parameter $\alpha \in {𝕫}_{n}^{*}$ and estimates $\omega_{1},\;\omega_{2},\;\omega_{3}$ as follows:

$$\omega_{1}=T_{\alpha }^{a}\left({TD}_{C}\right)(mod\;n)$$

$$\omega_{2}=\left(\alpha \times T+\omega_{1}\right)(mod\;n)$$

$$\omega_{3}=T_{\omega_{1}\times d}^{a}\left(TD_{\mathrm{GM}}\right)(mod\;n)$$

The GM sends $\left(\omega_{1},\;\omega_{2},\;\omega_{3}\right)$ to the signcrypter after measuring all of the values.

Step 3. The signcrypter then uses this equation to check the parameter’s authenticity.

$$T_{\omega_{2}}^{a}\left({TD}_{C}\right)=\left(T_{T}^{a}\left(\omega_{1}\right)\times T_{eW}^{a}\left(\omega_{3}\right)\right)(mod\;n)$$

If this equation holds true, the client will receive three factors; if it does not, the client will return it to the GM.

Correctness.

$$\begin{array}{rl} & T_{\omega_{2}}^{a}\left(TD_{C}\right)=\left(T_{\alpha T}^{a}\left(TD_{C}\right)\times T_{\omega_{1}}^{a}\left(TD_{C}\right)\right)(mod\;n)\\ & \;=\left(T_{T}^{a}\left(\omega_{1}\right)\times T_{W\omega_{1}}^{a}\left(TD_{\mathrm{GM}}\right)\right)(mod\;n)\\ & \;=\left(T_{T}^{a}\left(\omega_{1}\right)\times T_{\left(\frac{W}{d}\right)}^{a}\left(\omega_{3}\right)\right)(mod\;n)\\ & \;=\left(T_{T}^{a}\left(\omega_{1}\right)\times T_{eW}^{a}\left(\omega_{3}\right)\right)(mod\;n) \end{array}$$

### 4.5. Signcryption

The client will signcrypt the text on behalf of the party at this point. The client initially chooses a ᶇ∈ ${𝕫}_{n}^{*}$ private factor, after which he/she determines the following: Key (ƙ) and cipher (ƈ).

$$U=ᶇ+T_{\left(\frac{e}{\omega_{1}}\right)}^{a}\left(\omega_{3}\right)\left(mod\;n\right)$$

Key(ƙ)=ɦ(U×ᶇ)(mod n)

$$Cipher\;\left(ƈ\right)=\left(ƙ\times Message\left(m\right)\right)+G_{pbk}\left(mod\;n\right)$$

(3) $$\lambda =\left(T_{\omega_{3}}^{a}\left(G_{pbk}\right)\times T_{W}^{a}\left(TD_{\mathrm{GM}}\right)\right)\left(mod\;n\right)$$

(4) $$\;\lambda_{1}=T_{\omega_{3}}^{a}\left(g\right)\left(mod\;n\right)$$

(5) $$\lambda_{2}=\lambda +T_{m}^{a}\left(\lambda_{1}\right)\left(mod\;n\right)$$

The client then refers the verifier to the signcrypted text $\left(U,\;ƈ,\;\lambda ,\lambda_{1},\lambda_{2}\right)$.

### 4.6. Verification

The verifier confirms the legitimacy of the signcrypted information after discovering it, but first, they must locate the message. The verifier evaluates the following processes to locate a message:

$$ᶇ=\left(U-TD_{\mathrm{GM}}\right)\;\left(mod\;n\right)$$

(6) ƙ′=ɦ(U×ᶇ′) (mod n)

(7) $$m\prime =\left(U-G_{pbk}\right)\times {\left({ƙ}^{\prime }\right)}^{-1}\left(mod\;n\right)$$

Otherwise, they would dismiss the communication as illegitimate. As soon as the message is identified, the verifier verifies its legitimacy.

(8) $$\lambda_{2}=\lambda +T_{m}^{a}\left(\lambda_{1}\right)\left(mod\;n\right)$$

If this occurs, the verifier will create the signcrypted text on the message.

### 4.7. Opening

The GM will identify the sender if the sender is involved in a legal issue.

(9) $$TD_{C}=\frac{\lambda }{T_{prk}^{a}\left(\lambda_{1}\right)}\left(mod\;n\right)$$

## 5. Security Investigation of the Proposed CGSS-CCM ECS Scheme

The proposed CGSS-CCM scheme is given a formal security foundation in this section. As a result, two types of adversaries are studied, and the proposed technique’s security assessment is detailed as follows.

**Theorem** **1.** The CGSS-CCM generated signcrypted text that is correct.

**Proof.**  This theorem demonstrates the correctness property of the projected CGSS-CCM scheme. □

We can observe, as a result of Equation (5), that

$$ᶇ\prime =\left(U-TD_{\mathrm{GM}}\right)\left(mod\;n\right)\;\;=U-T_{ed}^{a}\left(TD_{\mathrm{GM}}\right)\left(mod\;n\right)\;\;=U-T_{{\left(ed\right)}^{\frac{\omega_{1}}{\omega_{3}}}}^{a}\left(TD_{\mathrm{GM}}\right)\left(mod\;n\right)\;\;=U-T_{\left(\frac{e}{\omega_{1}}\right)}^{a}\left(\omega_{3}\right)\left(mod\;n\right)\;\;=ᶇ$$

The suggested CGSS-CCM scheme appears to be implemented appropriately.

**Theorem** **2.** The CGSS-CCM is expected to have traceability capabilities, such as the ability for the GM only to open the signcrypter identification that has signed the signcrypted document.

**Proof.**  As a result of Equation (7), we realize that a signcrypter’s identity can be retrieved as $TD_{U}=\omega /\omega_{1}{}^{G_{prk}}$. □

Let

$$\frac{\lambda }{T_{G_{prk}}^{a}\left(\lambda_{1}\right)}=\frac{T_{\omega_{3}}^{a}\left(G_{pbk}\right)\times T_{W}^{a}\left(TD_{\mathrm{GM}}\right)}{T_{\omega_{3}G_{prk}}^{a}\left(g\right)}\left(mod\;n\right)=TD_{C}\left(mod\;n\right)$$

As a result, the traceability properties of the proposed CGSS-CCM approach are fulfilled.

**Theorem** **3.** Using the CCM-CDHP, the given CGSS-CCM can withstand Type-II and Type-I attacks, as stated below.

**Definition** **4.** (Type I Attack). A foe $\left({Ƒ}_{1}\right)$ having access to the device will be unable to gain the master secret key. However, ${Ƒ}_{1}$ can generate a signcrypted text by substituting public keys, removing private and partial private keys.

**Proof.**  The game is played among the challenger (Ƈ) and the foe $\left({Ƒ}_{1}\right)$ and the challenger (Ƈ) in the Type-I attack. The steps outlined below are used to communicate between them. □

PPKG: When the challenger (Ƈ) requests it, the challenger (Ƈ) conducts the setup procedure to generate a KGC’s master private key and a public factor $\left(m_{pk}\right)\;$ corresponding to the KGC’s identification (TD), then transmits $\left(m_{pk}\right)$ to the foe $\left({Ƒ}_{1}\right)$.

Key generation (KG): In the KG stage, the challenger (Ƈ) evaluates a (λ) private value after learning the GM’s identification $\left(TD_{\mathrm{GM}}\right)$, then uses the private key and partial secrete key to estimate the GM’s private key $\;\left(G_{prk}\right)$ and communicate it to the foe.

Request public key: For any identification, the adversary will now turn to the public key. The challenger calculates the value of the GM’s public key $\left(G_{prk}\right)$ and delivers it to the foe after getting the appeal.

Replace public key: The foe creates a novel $\lambda_{1}$ private value and substitutes the challenger’s public key with their own public key $\left(G_{prk1}\right)$ after obtaining the challenger’s public key.

Signcryption: For signcrypt, the client chooses specific secret values, but for a challenger message, the GM’s public key and the original text are required. The challenger then sends the signed text $S=\left(U,\;Ƈ,\lambda ,\;\lambda_{1},\;\lambda_{2}\right)$ on message $m_{1}$ to the foe using a public key for the sender’s identity that matches the GM’s public key. The foe wins the game if Designcrypt $\left(m_{pk1},\;TD_{\mathrm{GM}1},\;\lambda_{1},\;m_{1},\;S_{1}\right)$ equals 1, but the adversary does not breach the security since the foe cannot enquire about the signcryption on the message $m_{1}$ and the private key for an $TD_{\mathrm{GM}1}$.

**Definition** **5.** (Type II Attack). The foe $\left({Ƒ}_{2}\right)$ has retrieved the master key via a Type-II attack but cannot substitute any client’s public key.

**Proof.**  The challenger (Ƈ) and the foe $\left({Ƒ}_{2}\right)$ compete in this game. □

PPKG: The challenger then uses the setup method to generate a KGC’s master private key and an $\left(m_{pk}\right)$ public factor based on the KGC’s identity (TD), and then delivers the public and private keys to the foe. After that, the adversary would be able to estimate the partial private key.

Key generation: Following the GMs identify $\left(TD_{\mathrm{GM}}\right)$, the challenger (Ƈ) estimates a (λ) hidden value, calculates the GM’s private key $\left(G_{prk}\right)$ using the partial private key and secret key and delivers it to the foe $\left({Ƒ}_{2}\right)$.

Request public key: The challenger then determines the GM’s following public key and, upon request, provides it to the foe.

Signcryption: The challenger can now estimate a signcrypted text $S_{1}=\left(U,\;Ƈ,\lambda ,\;\lambda_{1},\;\lambda_{2}\right)$ on message $m_{1}$ and give it to the foe $\left({Ƒ}_{2}\right)$ using a public key for the sender’s identity and the GM’s public key. The foe wins the game if Designcrypt $\left(m_{pk1},\;TD_{\mathrm{GM}1},\;{Ѵ}_{1},\;m_{1},\;S_{1}\right)$ equals 1, but the adversary does not breach the security since the foe cannot request the signcryption on the message $m_{1}$ and the private key for an $\;TD_{\mathrm{GM}1}$. The presented system has also been proved to be resistant to Type-II and Type-I attacks.

**Theorem** **4.** The proposed CGSS-CCM satisfies the unlinkability property.

**Proof.**  The verifier confirms the signcrypted info by using the group’s $G_{pbk}\;$ public info and $\;TD_{\mathrm{GM}}$ as exposed in Equation (6) after discovering the group signcrypted info $(U,\;Ƈ,\lambda ,\;\lambda_{1},\;\lambda_{2}$) for m message .  If the verifier receives alternative signcrypted information $\left(U\prime ,\;Ƈ\prime ,\;\lambda \prime ,\;\lambda_{1}^{\prime },\;\lambda_{2}^{\prime }\right)$ for the message m′. In the two signcrypted info $\left(U,\;Ƈ,\lambda ,\;\lambda_{1},\;\lambda_{2}\right)$, there are no identical variables. When the verifier wishes to know the signcrypter’s identity (TD), they must consult the GM. The projected CGSS-CCM also comprises five variables, namely (α,ᶇ, U, W, e), to hide the precise estimate of the group’s signcrypted info/text. As a result, it is impossible to decode the estimates of (α,ᶇ, U) from the signcrypted data. As a result, an adversary would never be able to link signed data to the compliant signcrypter. □

## 6. Proposed Electronic Cash System Based on CGSS Using Conformable Chaotic Maps

**Figure 2:**
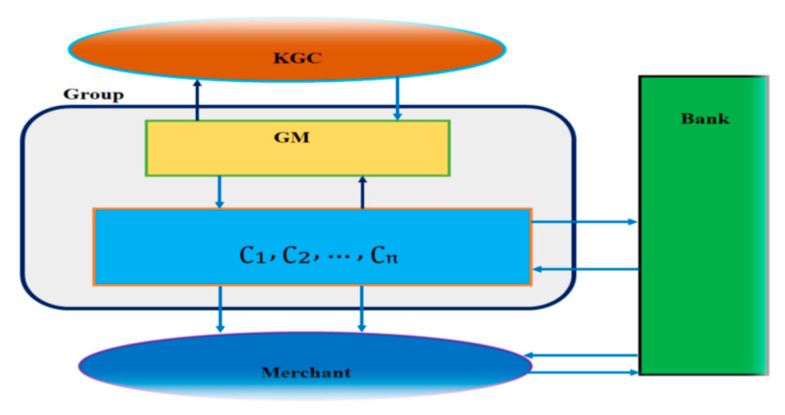
A model of the proposed electronic cash system (ECS).

This section proposes a new efficient electronic cash system based on CGSS using conformable chaotic maps. A consumer, a GM of that customer group (CG), a bank and a merchant participate in an ECS consisting of a series of protocols. In sum, an electronic cash system comprises the following six distinct phases, and Figure 2 depicts the planned E-cash scheme’s configuration.

### 6.1. Initialization

This stage is handled by a trusted third-party key generation center (KGC) and the GM because our projected method is a certificateless scheme. With KGC, the group’s GM establishes a public and private key for the group.

Step 1. The KCG selects an integer $n=p_{1}\times p_{2}$ where $p_{1},\;p_{2}$ are huge primes using the secure prime schemes. Then they select g as a GF $\left(p_{1}\right)$ generator and select a random a∈[0,1] rational number. Then they give g and n to the GM.

Step 2. The KGC selects two secret parameters $m_{sk}^{\prime }$ and $TD_{\mathrm{KGC}}^{\prime }\in {𝕫}_{n}^{*}$ at random and computes a public parameter $m_{pk}^{\prime }$ as a result.

(10) $$m_{pk}^{\prime }=T_{m_{sk}^{\prime }}^{a}\left(g\right)\left(mod\;n\right)$$

Then, on a secret channel, they send $\left(n,m_{pk}^{\prime },g\right)$ to the GM.

Step 3. The GM first selects a secret parameter $\lambda \prime \in \;{𝕫}_{n}^{*}$ and their identification as $TD_{\mathrm{GM}}^{\prime }$ after obtaining the parameter from the KGC, and then calculates the group’s public and private key as

$$G_{prk}^{\prime }=\lambda \prime \times m_{pk}^{\prime }+TD_{\mathrm{KGC}}^{\prime }\times TD_{\mathrm{GM}}^{\prime }\;\left(mod\;n\right)\;G_{Pbk}^{\prime }=T_{G_{prk}^{\prime }}^{a}\left(g\right)\left(mod\;n\right)\;e\equiv 1\;mod\;\phi \left(n\right).$$

The GM then makes $\left(n,\;g,TD_{\mathrm{GM}}^{\prime },\;e,\;G_{Pbk}^{\prime }\right)$ public to everybody while keeping $\left(\;e,\;\lambda \prime ,G_{prk}^{\prime }\right)$ private.

### 6.2. Joining Phase

Each customer ${ℭ}_{i}$ wishes to join the CG in this step. Thus they engage with the GM as in Step 1. Initial, each customer ${ℭ}_{i}$ selects a secret parameter $W\prime \in {𝕫}_{n}^{*}$ at random and calculates the subsequent:

(11) $$TD_{C_{i}}=T_{w\prime }^{a}\left({TD}_{\mathrm{GM}}^{\prime }\right)(mod\;n)$$

They then send it to the GM.

GM produces a membership certificate for each customer after determining their identification.

(12) $$m_{C_{i}}=T_{W\prime }^{a}\left(TD_{C_{i}})(mod\;n\right)$$

After, the GM adds a new record for customer identification with the membership certificate as $\left(m_{{ℭ}_{i}},\;d\right).$

### 6.3. Withdrawal Phase

The customer approaches the bank and requests a coin. The bank demands identity confirmation from the customer; thus, the customer must complete the promise stage before the procedure can be signcrypted.

Step 1. In this stage, each customer selects two secret parameters at random: $\alpha^{\prime },{ᶇ}^{\prime }\in \;{𝕫}_{n}^{*},$ and calculates the signcrypted text as follows:

$$\omega_{11}=T_{\alpha^{\prime }}^{a}\left(TD_{C_{i}}\right)(mod\;n)$$

$$\omega_{21}=\alpha^{\prime }\times T+\omega_{11}(mod\;n)$$

where T is the time and date concatenation.

$$\omega_{31}=T_{\omega_{11}\times e}^{a}\left(m_{C_{i}}\right)(mod\;n)$$

$$U^{\prime }={ᶇ}^{\prime }+TD_{\mathrm{GM}}^{\prime }(mod\;n)$$

$$\mathrm{Key}\left({ƙ}^{\prime }\right)=ɦ\left(U^{\prime }\times {ᶇ}^{\prime }\right)(mod\;n)$$

$$C^{\prime }=k^{\prime }\times m^{\prime }+G_{Pbk}^{\prime }(mod\;n)$$

Step 2. The bank verifies the text’s legitimacy after discovering the signcrypted text from the customer.

$$T_{\omega_{21}}^{a}\left(TD_{C_{i}}\right)=T_{T}^{a}\left(\omega_{11}\right)\times \omega_{31}(mod\;n)$$

Correctness.


$$T_{\omega_{21}}^{a}\left(TD_{C_{i}}\right)=T_{\alpha^{'}\times T+\omega_{11}}^{a}\left(TD_{C_{i}}\right)(mod\;n)\;=T_{\alpha^{'}\times T}^{a}\left(TD_{C_{i}}\right)\times T_{\omega_{11}}^{a}\left(TD_{C_{i}}\right)(mod\;n)\;=T_{T}^{a}\left(\omega_{11}\right)\times T_{\omega_{11}/d}^{a}\left(m_{C_{i}}\right)(mod\;n)\;=T_{T}^{a}\left(\omega_{11}\right)\times T_{\omega_{11}\times e}^{a}\left(m_{C_{i}}\right)(mod\;n)\;=T_{\omega_{21}}^{a}\left(TD_{C_{i}}\right)=T_{T}^{a}\left(\omega_{11}\right)\times \omega_{31}(mod\;n)$$

If this equation is true, the bank calculates

$$\begin{array}{c} \xi^{\prime }=T_{\omega_{31}}^{a}\left(G_{Pbk}^{\prime }\right)\times TD_{C_{i}}(mod\;n)\\ \xi_{1}^{\prime }=T_{\omega_{31}}^{a}(g)(mod\;n) \end{array}$$

The bank then sends the consumer these two parameters $\left(\xi \prime ,\;\xi_{1}^{\prime }\right)$ as their bank identification.

Step 3. The customer calculates another secret parameter after obtaining the secret parameter from the bank.

$$\xi_{3}^{\prime }=T_{n\prime }^{a}\left(\xi_{2}^{\prime }\right)(mod\;n)$$

where $\xi_{2}^{\prime }=\left(\xi^{\prime }+\xi_{1}^{\prime }\right)\;$ and stores the coin as $\left(\xi_{3}^{\prime },ƙ\prime ,\;ℭ\prime ,\;U\prime \right)$

### 6.4. Payment Phase

The interaction between the merchant and the customer takes place during this phase.

Step 1. The customer delivers the coin $\left(\xi_{2}^{\prime },\;\xi_{3}^{\prime },ƙ\prime ,\;ℭ\prime ,\;U\prime \right)$ to the merchant for payment. After locating the coin, the merchant first validates its legitimacy, which requires them to compute.

$$ᶇ\prime =\;U\prime -TD_{\mathrm{GM}}\prime \left(mod\;n\right).$$

Then they determine if the condition’s value is met or not.

ɦ(U′×ᶇ′)=ƙ′(mod n),

If yes, the merchant proceeds to the next step; otherwise, the customer is notified.

$$₣=ℭ\prime -G_{Pbk}^{\prime }\left(mod\;n\right)$$

and F’s value is sent to the consumer.

Step 2. The customer then generates a new parameter as follows:

₣′=(₣/m′) (mod n)

and ₣′ value is sent to the merchant. If ₣′=ƙ′, the merchant accepts the coin.

### 6.5. Deposit Phase

The interaction between the bank and the merchant is described in this phase.

Step 1. The merchant transmits this signcrypted text $\left(\xi_{2}^{\prime },\;\xi_{3}^{\prime },\;ƙ\prime ,\;ℭ\prime ,\;U\prime \right)$ and the coin (₣′,₣) to the bank after accepting the coin.

Step 2. The bank checks whether ɦ(U′×ᶇ′)=ƙ′(mod n) if the coin exists, otherwise it sends an incorrect message.

The bank stores the coin (₣′, ₣) in the placed table if it is valid.

### 6.6. Identity Revocation Phase

In the event of a dispute, the bank will submit the signcrypted document to the GM, who will then identify the dishonest customer.

(13) $$TD_{{ℭ}_{i}}=\xi \prime /T_{G_{prk}^{\prime }}^{a}\left(\xi_{1}^{\prime }\right)\;\left(mod\;n\right)$$

## 7. Security Analysis of the Proposed CGSS-CCM ECS Scheme

This section details some of the security and effectiveness features of our ECS scheme. We demonstrate that our offline ECS scheme is secure from threats, such as forgery and anonymity.

### 7.1. Unforgeability

In the suggested approach, a fraudulent customer cannot falsify the coin because, in the event of blackmail or a legal disagreement, the bank notifies the GM of that client group. The GM can then use the equation $TD_{{ℭ}_{i}}=\xi \prime /T_{G_{prk}^{\prime }}^{a}\left(\xi_{1}^{\prime }\right)\left(mod\;n\right)$ to identify the customer’s identification, and only the user who is the account owner in the withdrawal protocol can withdraw an e-coin.

### 7.2. Anonymity

The projected technique allows the user to make an anonymous payment to the merchant because the retailer is unaware of the customer’s identity. They can only accept a coin from the user and check the correctness of the signcrypted document, but the merchant has no way of knowing who the customer is. As a result, the suggested system is unaffected by the anonymity attribute.

## 8. Performance Comparison

**Figure 3:**
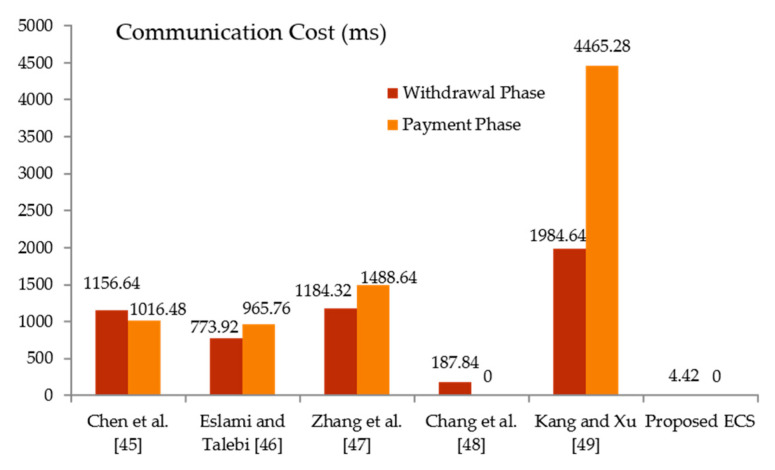
Communication cost (ms) in withdrawal and payment phases.

**Figure 4:**
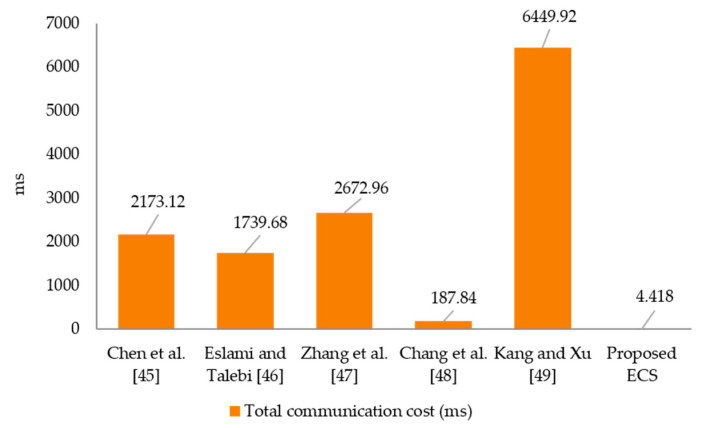
Total communication cost (ms).

In this section, we compare our technique to recently contributed electronic cash systems [45,46,47,48,49] in terms of communication cost. The efficiency of the provided electronic cash system is evaluated based on communication costs. The output is compared based on the cost of the withdrawal and payment phases. In contrast to the installation, joining, deposit, and identity revocation stages, the withdrawal and payment phases need additional computational resources. As a result, the computation cost for the withdrawal and payment phases is used to perform the comparison analysis. In this part of the comparisons study, we utilized the following six notations of this complexity: ${Ϯ}_{h},{Ϯ}_{m},\;{Ϯ}_{ch},$ ${Ϯ}_{e}$, ${Ϯ}_{sy\;},{Ϯ}_{ec}\;$ and ${Ϯ}_{p}$ reported performance time for a one-way hash function modular multiplication, Chebyshev chaotic map operation, modular exponentiation in the group, symmetric encryption operation, elliptic curve scale multiplication, and bilinear pairing operations. The relations among ${Ϯ}_{h},{Ϯ}_{m},\;{Ϯ}_{ch},$ ${Ϯ}_{e}$, ${Ϯ}_{sy\;},{Ϯ}_{ec}\;$ and ${Ϯ}_{p}$ with respect to $\;{Ϯ}_{h}$ $({Ϯ}_{h}=0.32\;\mathrm{ms}$ and a=1/2 since a∈[0,1] [46]) have been established in [38,50,51,52,53]. The following illustration depicts the relationship and order of computational complexity between the metrics:$\;{Ϯ}_{ch}\approx {Ϯ}_{h},{Ϯ}_{m}\approx 2.5\;{Ϯ}_{h},\;{Ϯ}_{sy\;}\approx {Ϯ}_{h},{Ϯ}_{ec}\approx 72.5{Ϯ}_{h},\;{Ϯ}_{e}\approx 600{Ϯ}_{h}$,$\;{Ϯ}_{p}\approx 1550{Ϯ}_{h}\;$ and ${Ϯ}_{h}\approx {Ϯ}_{ch}\approx {Ϯ}_{sy}<{Ϯ}_{m}<{Ϯ}_{ec}<{Ϯ}_{e}<{Ϯ}_{p}$. Table 1 depicts the predicted electronic cash system’s primary consuming operations as well as existing techniques. There are additional comparisons of computing costs in milliseconds (ms) in Figure 3. The evaluation results in Table 2 and Figure 4 show that the suggested electronic cash system has the lowest overall communication expense. In terms of running time, the proposed electronic cash system outperforms the other methods. The proposed CGSS-CCM ECS scheme would find useful applications in emerging wireless communication systems in the 6G era and beyond [54].

## 9. Conclusions

This paper proposed an efficient and effective ECS based on the concept of CGSS-CCM, which is secure against an IND-CCA attack in conformable chaotic maps. In order to demonstrate the strengths of our CGSS-CCM enabled scheme, we performed standard security examinations. We found that it meets the requirements for anonymity and unforgeability in a well-designed and secure electronic cash payment system. Additionally, we compared the computational costs of our scheme with five other schemes, and the results showed that our ECS had lower costs than the other five schemes. Finally, our scheme can be helpful in many real-life applications, such as online auctions, e-banking, and electronic voting systems. Future work could extend the proposed CGSS-CCM assisted scheme to ease its applicability in emerging wireless application scenarios.

## Figures and Tables

**Table 1:** List of symbols.

Symbol	Meaning
$T^{a}$	Conformable Chebyshev chaotic maps
n	Large integer
$p_{1},p_{2}$	Large prime numbers
$TD_{\mathrm{KGC}}$	Identity of KGC
$TD_{\mathrm{GM}}$	Identity of GM
$TD_{C}$	Identity of C client
a	An arbitrary rational number
$m_{sk}$	Master secret key
$G_{prk}$	Group’s public key
$G_{pbk}$	Group’s private key
ƈ	Cipher
ɦ	Hash function
$m_{pk}$	Public constraint
m	Message
ƙ	Key

**Table 2:** Assessments of important operations with reverence techniques.

Techniques	Withdrawal Phase	Payment Phase	Total
Chen et al. [45]	$3{Ϯ}_{h}+3{Ϯ}_{p}+4{Ϯ}_{sy\;}+7{Ϯ}_{ec}$	$2{Ϯ}_{h}+3{Ϯ}_{p}+2{Ϯ}_{sy\;}+{Ϯ}_{ec}$	$5{Ϯ}_{h}+6{Ϯ}_{p}+6{Ϯ}_{sy\;}+8{Ϯ}_{ec}$
Eslami and Talebi [46]	$4{Ϯ}_{e}+7{Ϯ}_{m}+{Ϯ}_{h}$	$5{Ϯ}_{e}+6{Ϯ}_{m}+3{Ϯ}_{h}$	$9{Ϯ}_{e}+13{Ϯ}_{m}+4{Ϯ}_{h}$
Zhang et al. [47]	$2{Ϯ}_{h}+2{Ϯ}_{p}+{Ϯ}_{e}$	$2{Ϯ}_{h}+3{Ϯ}_{p}$	$4{Ϯ}_{h}+5{Ϯ}_{p}+{Ϯ}_{e}$
Chang et al. [48]	$3{Ϯ}_{h}+4{Ϯ}_{sy\;}+8{Ϯ}_{ec}$	0	$3{Ϯ}_{h}+4{Ϯ}_{sy\;}+8{Ϯ}_{ec}$
Kang and Xu [49]	$2{Ϯ}_{h}+4{Ϯ}_{p}$	$4{Ϯ}_{h}+9{Ϯ}_{p}$	$6{Ϯ}_{h}+13{Ϯ}_{p}$
Proposed ECS	$3{Ϯ}_{ch}+{Ϯ}_{h}+3{Ϯ}_{m}$	0	$3{Ϯ}_{ch}+{Ϯ}_{h}+3{Ϯ}_{m}$

## Data Availability

Data sharing is not applicable to this article.
